# Expressing a Target Mimic of miR156fhl-3p Enhances Rice Blast Disease Resistance Without Yield Penalty by Improving *SPL14* Expression

**DOI:** 10.3389/fgene.2020.00327

**Published:** 2020-04-23

**Authors:** Ling-Li Zhang, Yan Li, Ya-Ping Zheng, He Wang, Xuemei Yang, Jin-Feng Chen, Shi-Xin Zhou, Liang-Fang Wang, Xu-Pu Li, Xiao-Chun Ma, Ji-Qun Zhao, Mei Pu, Hui Feng, Jing Fan, Ji-Wei Zhang, Yan-Yan Huang, Wen-Ming Wang

**Affiliations:** ^1^Rice Research Institute and Key Lab for Major Crop Diseases, Sichuan Agricultural University at Wenjiang, Chengdu, China; ^2^State Key Laboratory of Crop Gene Exploration and Utilization in Southwest China, Sichuan Agricultural University at Wenjiang, Chengdu, China

**Keywords:** microRNA, osa-miR156, rice blast disease resistance, *Magnaporthe oryzae*, *SPL14*, *WRKY45*

## Abstract

MicroRNAs (miRNAs) play essential roles in the regulation of plant growth and defense responses. More and more, miRNA-3ps are reported to act in plant development and immunity. miR156 is a conserved miRNA, and most previous studies focus on its roles in plant growth, development, and yield determinacy. Here, we show that expressing a target mimic of miR156fhl-3p led to enhanced rice blast disease resistance without a yield penalty. miR156fhl-3p was differentially responsive to *Magnaporthe oryzae* in susceptible and resistant accessions. Transgenic lines expressing a target mimic of miR156fhl-3p (MIM156-3p) exhibited enhanced rice blast disease resistance and increased expression of defense-related genes. MIM156-3p also enhanced the mRNA abundance of *SPL14* and *WRKY45* by down-regulating miR156-5p and pre-miR156. Moreover, MIM156-3p lines displayed a decreased number of second rachis branches per panicle but enlarged grains, leading to unchanged yield per plant. Consistently, overexpressing miR156h (OX156) led to enhanced susceptibility to *M. oryzae* and decreased the expression of *SPL14* and *WRKY45*. Our results indicate that miR156fhl-3p mounts a regulatory role on miR156-5p, which subsequently regulates the expression of *SPL14* and *WRKY45* to improve rice blast disease resistance.

## Introduction

MicroRNAs (miRNAs) are a kind of 20- to 24-nucleotide (nt) non-coding RNA molecule that regulate the expression of genes with sequences complementary to the miRNAs (Bartel, 2004; Yu et al., 2017). One miRNA isoform can be transcribed from one or more *MIR* gene loci (Li et al., 2019). A *MIR* gene is first transcribed into a pri-miRNA, which is processed by Dicer-like proteins (DCLs) to form pre-miRNA (Rogers and Chen, 2013). The pre-miRNA is further cleaved by DCLs, generating a miRNA-5p/miRNA-3p (previously miRNA/miRNA^∗^) duplex (Liu et al., 2005). Then, the duplex separates into a mature miRNA arm (-5p or -3p) that is bound by Argonaute (AGO) proteins to form RNA-induced silencing complex (RISC) (Ji et al., 2011; Zhu et al., 2011; Kobayashi and Tomari, 2016). RISC binds to the sequences reversely complementary to the miRNA and triggers DNA methylation, mRNA cleavage, or translational inhibition, respectively (Iwakawa and Tomari, 2015; Yu et al., 2017). The selection of a mature miRNA (-5p or -3p arm) can be different across tissues, developmental stages, and abiotic or biotic stresses (Guo et al., 2014; Hu et al., 2014). Some *MIR* genes are only processed into miRNA-5p, and some miRNAs are processed into miRNA-5p and miRNA-3p. The miRNA-3p was once considered as a useless by-product of the miRNA biogenesis. Some recent studies showed that a miRNA-5p and a miRNA-3p could target different gene families, each of which is involved in diverse biological processes (Jones-Rhoades et al., 2006; Huang et al., 2019). For example, in *Arabidopsis*, miR393 can be induced by a well-studied pathogen-associated molecular pattern (PAMP) molecule, flg22, and positively contributes to resistance against *Pseudomonas syringae* pv. *tomato* DC3000 (*Pst* DC3000) via miR393-5p-mediated suppression of the expression of *Transport Inhibitor Response 1* (*TIR1*), *Auxin signaling F-Box proteins 2* (*AFB2*), and *AFB3* (Navarro et al., 2006). On the other hand, miR393-3p targets *MEMB12*, a gene encoding a Gogi-localized SNARE (soluble N-ethylmaleimide-sensitive factor attachment protein receptor) protein involved in protein trafficking and regulating plant immune responses (Zhang et al., 2011). However, it is unclear whether the accumulation of a -3p correlates with a -5p.

Rice feeds half of the world population. Rice production is continually threatened by blast disease that is caused by *Magnaporthe oryzae* (syn. *Pyricularia oryzae*) (Zhang et al., 2018). In the past decade, more than 70 miRNAs have been identified to be responsive to *M. oryzae* or its elicitors, and 11 of them have been functionally characterized to play positive or negative roles in rice blast disease resistance (Li et al., 2019; Zhou et al., 2019). For example, miR160 positively regulates rice immunity against *M. oryzae* via up-regulating defense-related gene expression and hydrogen peroxide accumulation at the infection site (Li et al., 2014). miR167d facilitates *M. oryzae* infection by repressing the expression of *Auxin Responsive Factor 12* (*ARF12*) (Zhao et al., 2019). miR169 negatively regulates rice immunity by repressing *Nuclear Factor Y-A* (*NF-YA*) genes (Wu et al., 2009; Li et al., 2017). miR319 targets the transcription factor *Teosinte Branched1/Cycloidea/Proliferating Cell Factor1* (*OsTCP21*), which manipulates JA synthesis via *OsLOX2* and *OsLOX5*, the key synthetic components of JA, to facilitate the blast fungus infection (Hao et al., 2012; Zhang et al., 2018). miR396 targets *Growth Regulating Factor* (*GRF*) genes to balance growth, yield traits, and immunity against *M. oryzae* (Chandran et al., 2019). Different miR156-3p isoforms are identified to be responsive to the infection of *M. oryzae* (Li et al., 2014), but their roles are unknown.

miR156 is a conserved and highly abundant miRNA in most plants and acts as a master regulator during the whole growth period (Axtell and Bowman, 2008). In *Arabidopsis*, miR156 is highly expressed in young seedlings and miR156-5p target *SQUAMOSA promoter-binding protein-like transcription factor* (*SPL*) genes to regulate growth and development (Cardon et al., 1999; Wu and Poethig, 2006; Addoquaye et al., 2008; Shikata et al., 2009; Xu et al., 2016). These *Arabidopsis SPL* genes can be classified into three functionally distinct groups. The first group includes *SPL2*, *SPL9*, *SPL10*, *SPL11*, *SPL13*, and *SPL15*, which regulate juvenile-to-adult vegetative and vegetative-to-reproductive transition. The second group includes *SPL3*, *SPL4*, and *SPL5*, which could promote the floral meristem identity transition. The third group is *SPL6*, which may be important for physiological processes (Xie et al., 2006; Riese et al., 2007; Preston and Hileman, 2013). In rice, the *Osa-miR156* gene family has 12 members that generate three mature miR156-5p isoforms and four mature miR156-3p isoforms. miR156-5p targets 11 rice *SPL* genes that are involved in the regulation of important agronomic traits and blast disease resistance (Xie et al., 2006). For example, *SPL13* positively contributes to rice yield via improving grain size and panicle size (Si et al., 2016). *SPL16* positively regulates grain size via binding to *GW7*, which acts as a critical regulator in rice architecture and grain development (Wang et al., 2012; Wang S. et al., 2015). *SPL18* negatively regulates grain number through binding to the promoter of *DEP1* that acts as an important regulator of panicle architecture (Huang et al., 2009; Taguchi-Shiobara et al., 2011; Yuan et al., 2019). *SPL14* is also known as *Ideal Plant Architecture 1* (*IPA1*), which plays a critical role in rice architecture and resistance to *M. oryzae* via phosphorylation status and subsequent DNA-binding specificity (Jiao et al., 2010; Wang et al., 2018). Before *M. oryzae* invasion, SPL14 protein binds to the *DEP1* promoter that contains the GTAC motif, to regulate tiller development, leading to plants with fewer unproductive tillers and more grains per panicle, supporting higher yield (Wang et al., 2018). Upon *M. oryzae* invasion, SPL14 protein is phosphorylated and binds to the *WRKY45* promoter that contains TGGGCC motif, leading to enhanced resistance to the blast disease (Wang et al., 2018).

*WRKY45* encodes a WRKY transcription factor that binds to W-box or W-like box-type cis-elements in response to pathogens and plays a vital role in plant-pathogen interaction (Li et al., 2004; Eulgem and Somssich, 2007). Overexpression of *WRKY45* markedly enhances rice blast resistance, whereas knock out of *WRKY45* compromises blast resistance (Shimono et al., 2007). The *WRKY45* transcript responds strongly to SA and BTH, but its role is neither downstream nor upstream of *NH1*, a rice homolog of *NPR1*. In contrast, a glutathione S-transferase and a cytochrome P450 are found to be downstream of *WRKY45*. Elevated *WRKY45* mRNA level could remarkably enhance resistance to *M. oryzae* (Shimono et al., 2007; Goto et al., 2015, 2016). However, constitutive *WRKY45* overexpression-conferred blast resistance remarkably penalizes yield (Goto et al., 2015). Expressing *WRKY45* under the control of pathogen-responsive promoters in combination with a translational enhancer derived from a 5′-untranslated region (UTR) of rice *alcohol dehydrogenase* (*ADH*) has strong disease resistance without penalty yield (Goto et al., 2016). Therefore, it is highly anticipated that miR156 plays an essential role in the regulation of rice blast disease resistance via the *SPL14*-*WRKY45* module. However, experimental evidence is lacking, and whether miR156-3p plays roles in blast resistance is unclear.

To investigate the function of miR156-3p in rice blast disease resistance, we constructed the transgenic lines expressing a target mimic of miR156fhl-3p (MIM156-3p) and found that MIM156-3p showed enhanced blast disease resistance. Surprisingly, the MIM156-3p plants exhibited significantly less pre-miR156 and miR156-5p accumulation than control, indicating that blocking miR156fhl-3p interfered with the production of miR156-5p, which in turn led to increase in the expression of *SPL14* and *WRKY45*. In contrast, elevated miR156h expression (OX156) enhanced susceptibility to *M. oryzae*, which could be due to the suppression of *SPL14* and decreased *WRKY45* expression. Moreover, MIM156-3p plants displayed an unchanged yield per plant. Together, our data indicate that miR156flh-3p and miR156-5p may be mutually regulated via an unknown mechanism, and miR156 negatively regulates rice blast disease resistance via *SPL14* and *WRKY45*. Our results provide a potential tool to improve blast resistance without yield penalty and a new phenomenon in miRNA synthesis/accumulation.

## Materials and Methods

### Plant Materials and Growth Conditions

Rice (*Oryza sativa*) materials used in this study include Lijiangxin Tuan Heigu (LTH) and International Rice Blast Line Pyricularia-Kanto51-m-Tsuyuake (IRBLkm-Ts). LTH is a susceptible accession sensitive to over 1,300 regional isolates of *M. oryzae* worldwide (Lin et al., 2001). IRBLkm-Ts is a resistant accession, carrying a resistance locus *Pi-km* (Hiroshi et al., 2000). The rice cultivar Nipponbare was used as wild type (WT) and transgenic background. For germination, rice seeds were immersed in water for 2 d at 37°C in darkness. Then, seedlings were grown in an air-conditioned growth room at 26°C and 70% relative humidity with a 14 h light/10 h dark cycle. *Nicotiana benthamiana* plants were planted in an air-conditioned growth room at 24°C with a 16 h light/8 h dark cycle. *N. benthamiana* plants were used for *Agrobacterium*-infiltration experiments.

### Plasmid Construction and Genetic Transformation

The artificial target mimic of miR156fhl-3p was acquired by annealing with primers miR156-mimic-F and miR156-mimic-R (Supplementary Figure S1 and Supplementary Table S1). The fragments were inserted into *IPS1* (*INDUCED BY PHOSPHATE STARVATION1*) to replace the miR399 target site as described previously (Franco-Zorrilla et al., 2007; Wu et al., 2013). Then, the DNA fragments were cloned into *Bam*HI/*Bgl*II site of pCAMBIA1300-35S, resulting in overexpressing construct p35S:MIM156-3p. To generate transgenic plants overexpressing miR156h, we amplified the genomic sequences containing 487 bp upstream and 403 bp downstream of the *MIR156h* gene from Nipponbare genomic DNA with primers miR156h-F and miR156h-R (Supplementary Table S1). The amplified fragments were digested with *Kpn*I/*Sal*I. After purified by DNA Purification Kit (Invitrogen), the fragments were cloned into vector pCAMBIA1300, resulting in the overexpressing construct p35S:miR156h. The plasmids were introduced into the Nipponbare background via *Agrobacterium* (strain GV3101)-mediated transformation. Positive transformants were screened following a previous report (Li et al., 2014).

### Disease Assay and Microscopy Analysis

Three *M. oryzae* strains, including Zhong10-8-14-GFP (GZ8), 97-27-2, and FJ08-09-1, were used, depending on the availability at the time for inoculation. GZ8 and 97-27-2 were used for punch/spray-inoculation assay, and GZ8 was used for rice sheath inoculation assay in transgenic rice plants in Nipponbare background; FJ08-09-1 was incompatible with resistance accession IRBLkm-Ts and used for spray-inoculation assay in susceptible accession LTH and IRBLkm-Ts. For sporulation, *M. oryzae* strains were cultured in complete medium and oatmeal and tomato media (OTA) at 28°C with 16 h/8 h light/dark. For disease assays via spray-inoculation, the spores of the indicated strains were collected and adjusted to 1 × 10^5^ spores mL^–1^ to spray onto three-leaf-seedlings. The inoculated seedlings were incubated in darkness with 100% humidity for 24 h and then put in an air-conditioned growth room (Li et al., 2017). Leaves were collected at 12 and 24 h post-inoculation (hpi) for analysis on the expression of defense-related genes. For disease assays via punch-inoculation, the collected spores (3 × 10^5^ spores mL^–1^) were drop-inoculated on the punch-wound rice leaves and cultured in buffer containing 0.1% 6-Benzylaminopurine (6-BA). The leaves were incubated in darkness for 24 h, and then continually incubated in 100% humidity and 12 h/12 h light/dark conditions. The disease phenotypes via punch/spray-inoculation were recorded at 5 days post-inoculation (dpi) (Chandran et al., 2019; Zhao et al., 2019). Disease severity was measured by the relative fungal mass that was calculated using the DNA level of *MoPot2* against the rice *ubiquitin* gene via quantitative PCR (Li et al., 2017). For rice-sheath inoculation assays, 7 cm-long leaf sheaths were injected with GZ8 (1 × 10^5^ spores mL^–1^) spore suspension (Kankanala et al., 2007). The epidermal layer from the inoculated leaf sheaths was analyzed by Laser Scanning Confocal Microscopy (LSCM) (Nikon A1) at 24 and 36 hpi, respectively.

### Gene Expression Analyses

Total RNAs were extracted from rice leaves using TRIzol reagent (Invitrogen), according to the manufacturer’s instructions. The first-strand cDNA was synthesized by using Primescript RT reagent Kit with gDNA Eraser, following the manufacturer’s instructions [TaKaRa Biotechnology (Dalian) Co., Ltd., Japan]. RT-qPCR was performed by using specific primers and SYBR Green mix (SYBR Green PCR Kit, Bimake) with BIO-RAD C1000TM Thermal Cycler. Rice *ubiquitin* (*UBQ*) gene was used as an internal reference for data normalization. The total RNA was reverse-transcribed by using a specific stem-loop RT primer (miR156 stem-loop RT primer) to examine the expression of mature miR156. The RT products were subsequently used as the template for RT-qPCR by using a miR156-specific forward primer and a universal reverse primer. The stem-loop primers were designed as described previously (Chen et al., 2005). The snRNA U6 was used as an internal reference for the detection of miRNAs. RT-qPCR was carried out by using the indicated primers (Supplementary Table S1).

### Transient Expression Assay in *N. benthamiana*

The *Agrobacterium* strain GV3101 carrying the individual construct in the binary vector pCAMBIA1300 was incubated in LB media containing specific antibiotics (rifampin, 50 μg/mL; kanamycin, 50 μg/mL) at 28°C incubation. The bacteria were collected at 5,000 rpm for 5 min and resuspended in MMA buffer (10 mM MES, 10 mM MgCl_2_, and 100 μM AS). The *Agrobacteria* containing the expression constructs were infiltrated into leaves of *N. benthamiana* for transient expression assay. Leaves were examined for image acquisition by using the Zeiss fluorescence microscope (Zeiss imager A2) between 36 and 72 hpi. Western blot analyses were performed following a previous protocol (Li et al., 2017). Total proteins were extracted from equal amounts of leaves with the protein extraction buffer with 1 × loading buffer (0.05 mg/mL Bromophneil blue; 0.065 M Tris-HCl, pH 6.8; 0.02 g/mL SDS; 0.05 mL/mL 2-mercaptoethanol; 0.1 mL/mL glycerol; 1 × protease inhibitor cocktail EDTA-free, Bimake, b14002). Total proteins were separated by 10% SDS-PAGE and transferred to PVDF membrane (Millipore) by using Trans-Blot Turbo (BIO-RAD). Protein blot was hybridized with the rabbit anti-GFP to determine YFP accumulation, and membranes were stained with Ponceau S as the loading control.

### Agronomic Traits Measurement

The rice plants for agronomic traits assay were planted in a paddy field in Chengdu, China (36° N, 103° E), during the rice-growing season from April to September. Five representative plants for each line were sampled for measurement of agronomic traits, including plant height, number of tillers, panicle length, number of primary rachis branches (PBs) per panicle, number of secondary rachis branches (SBs) per panicle, number of seeds per panicle, seed length, seed width, 1,000-grain weight, and grain yield per plant on a SC-G seed-counting and grain-weighing device (Wanshen Ltd., Hangzhou, China). The data were analyzed by a one-way ANOVA followed by *post hoc* Tukey HSD analysis with significant differences (*P* < 0.05) by using SPSS statistics software.

## Results

### miR156fhl-3p Is Responsive to Chitin Treatment and *M. oryzae* Infection

**FIGURE 1:**
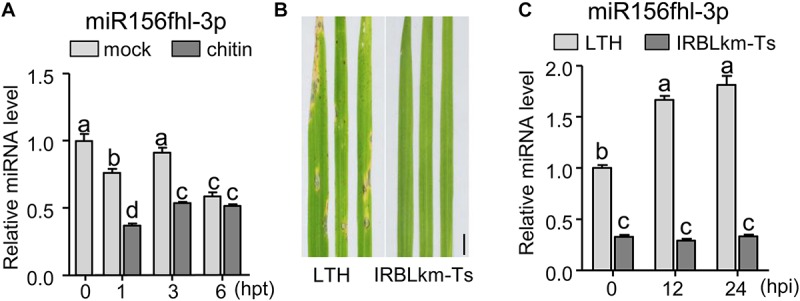
miR156fhl-3p is differentially responsive to chitin and *M. oryzae*. **(A)** Expression pattern of miR156fhl-3p upon chitin treatment. **(B)** Representative leaf sections from LTH and IRBLkm-Ts at 5 dpi upon *M. oryzae* strain FJ08-09-1. Scale bars, 1 cm. **(C)** Expression pattern of miR156fhl-3p in LTH and IRBLkm-Ts following *M. oryzae* inoculation. RNAs were extracted from leaves at the indicated time points. miR156fhl-3p was analyzed using Stem-loop RT-qPCR. SnRNA U6 served as the internal reference. Error bars indicate standard deviation (SD) (*n* = 3). Different letters above the bars indicate significant differences (*P* < 0.05) determined by one-way ANOVA analysis followed by *post hoc* Tukey HSD analysis. These experiments were repeated two times with similar results.

Rice genome contains twelve *MIR156* genes generating three mature miR156-5p isoforms and four miR156-3p isoforms (Supplementary Figure S1A)^1^. One major miR156-5p isoform was transcribed from *Osa-miR156a*, *Osa-miR156b*, *Osa-miR156c*, *Osa-miR156d, Osa-miR156e*, *Osa-miR156f*, *Osa-miR156g*, *Osa-miR156h*, *Osa-miR156i*, and *Osa-miR156j* (designated miR156-5p); whereas four miR156-3p isoforms were respectively transcribed from *Osa-miR156f*, *Osa-miR156h*, and *Osa-miR156l* (designated miR156fhl-3p), *Osa-miR156c* and *Osa-miR156g* (designated miR156cg-3p), *Osa-miR156b* (miR156b-3p), and *Osa-miR156j* (miR156j-3p) (Supplementary Figure S1A). In addition, *Osa-miR156f* and *Osa-miR156h* generated an identical pre-miRNA (designated pre-miR156fh). In our previous work, we detected the different expression patterns of miRNAs in resistance accession IRBLkm-Ts and susceptible accession LTH following the *M. oryzae* induction by high-through RNA-Seq assay. The abundance of miR156fhl-3p was remarkably higher than that of miR156b-3p, miR156cg-3p, and miR156j-3p, and all of their abundances were significantly lower in resistance accession IRBLkm-Ts than in susceptible accession LTH (Supplementary Figure S2; Li et al., 2014). Therefore, we focused on miR156fhl-3p in this study. To confirm miR156fhl-3p is responsive to the fungal pathogen, we conducted a time-course analysis of the accumulation pattern of miR156fhl-3p in LTH following chitin treatment, as well as in LTH and IRBLkm-Ts following infection of an *M. oryzae* strain FJ08-09-1 (Figure 1B). Chitin is a component of the fungal cell wall and can induce immune responses (Kawano and Shimamoto, 2013; Li et al., 2014; Kawasaki et al., 2017). Upon application of chitin, the abundance of miR156fhl-3p was significantly decreased at 1 and 3 h post-treatment (hpt) in comparison with mock treatment (Figure 1A), indicating that miR156fhl-3p is responsive to chitin treatment. In contrast, upon *M. oryzae* inoculation, the amount of miR156fhl-3p was significantly increased in susceptible accession LTH, and unchanged in IRBLkm-Ts at 12 and 24 hpi (Figure 1C). However, the amount of miR156fhl-3p was significantly less in resistance accession IRBLkm-Ts than that in susceptible accession LTH (Figure 1C). These results suggested that miR156fhl-3p is responsive to chitin and *M. oryzae*.

### Blocking miR156fhl-3p via a Target Mimic Compromises Rice Susceptibility to *M. oryzae*

**FIGURE 2:**
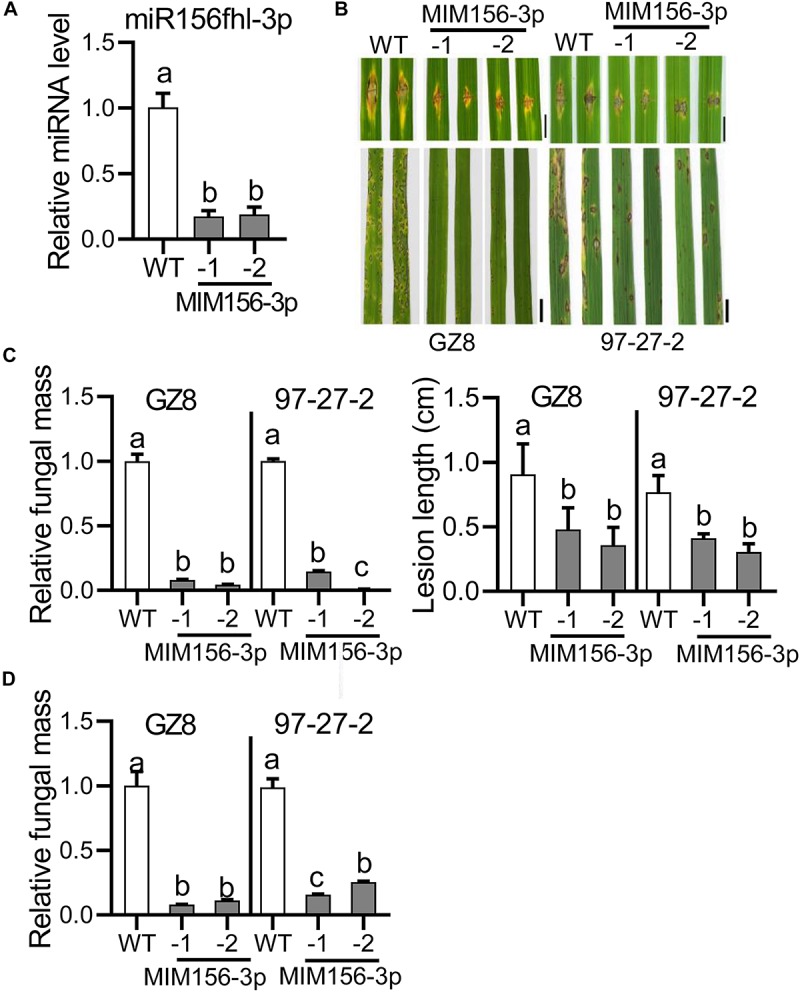
Expression of a target mimic of miR156fhl-3p leads to compromised susceptibility to *M. oryzae*. **(A)** Relative amounts of miR156fhl-3p in transgenic lines expressing a target mimic of miR156fhl-3p (MIM156-3p) and WT plants. The amounts of mature miR156fhl-3p were analyzed using stem-loop RT-qPCR. **(B)** Disease phenotype of control and MIM156-3p lines. Three-leaf-stage seedlings were punch/spray-inoculated with *M. oryzae* strains GZ8 and 97-27-2. Leaf phenotypes were observed at 5 dpi. Scale bars: Punch-inoculation assays are 5 mm; spray-inoculation assays are 1 cm. **(C)** Relative fungal mass represented by measuring *MoPot2* gene against *OsUbiquitin* DNA level and average lesion length via punch-inoculation. **(D)** Relative fungal mass via spray-inoculation. Error bars indicate SD (*n* = 3). Different letters above the bars indicate significant differences (*P* < 0.05) determined by one-way ANOVA analysis followed by *post hoc* Tukey HSD analysis. These experiments were repeated two times with similar results.

To detect the roles of miR156fhl-3p in rice resistance to blast disease, we constructed transgenic lines overexpressing a target mimic of miR156fhl-3p (MIM156-3p), which was complementary to miR156fhl-3p with 3-nt insertion between position 10 and 11 nts and acted as a sponge to hold miR156fhl-3p (Franco-Zorrilla et al., 2007; Supplementary Figure S1B). Out of seven lines, we selected two lines for further investigation, namely MIM156-3p-1 and MIM156-3p-2, which displayed significantly decreased miR156fhl-3p accumulation in comparison with control Nipponbare (WT) plants (Figure 2A). Both transgenic lines showed much smaller disease lesions and less fungal mass than control via punch-inoculation of the *M. oryzae* strain GZ8 and 97-27-2 (Figures 2B,C). Consistently, both transgenic lines displayed compromised susceptibility with smaller disease lesions and less fungal growth following spray-inoculation with GZ8 and another strain 97-27-2 (Figures 2B,D). These data indicate that blocking miR156fhl-3p compromises rice susceptibility to *M. oryzae*.

### Expression of a Target Mimic of miR156fhl-3p Delays *M. oryzae* Infection and Enhances the Induced Expression of Defense-Related Genes

**FIGURE 3:**
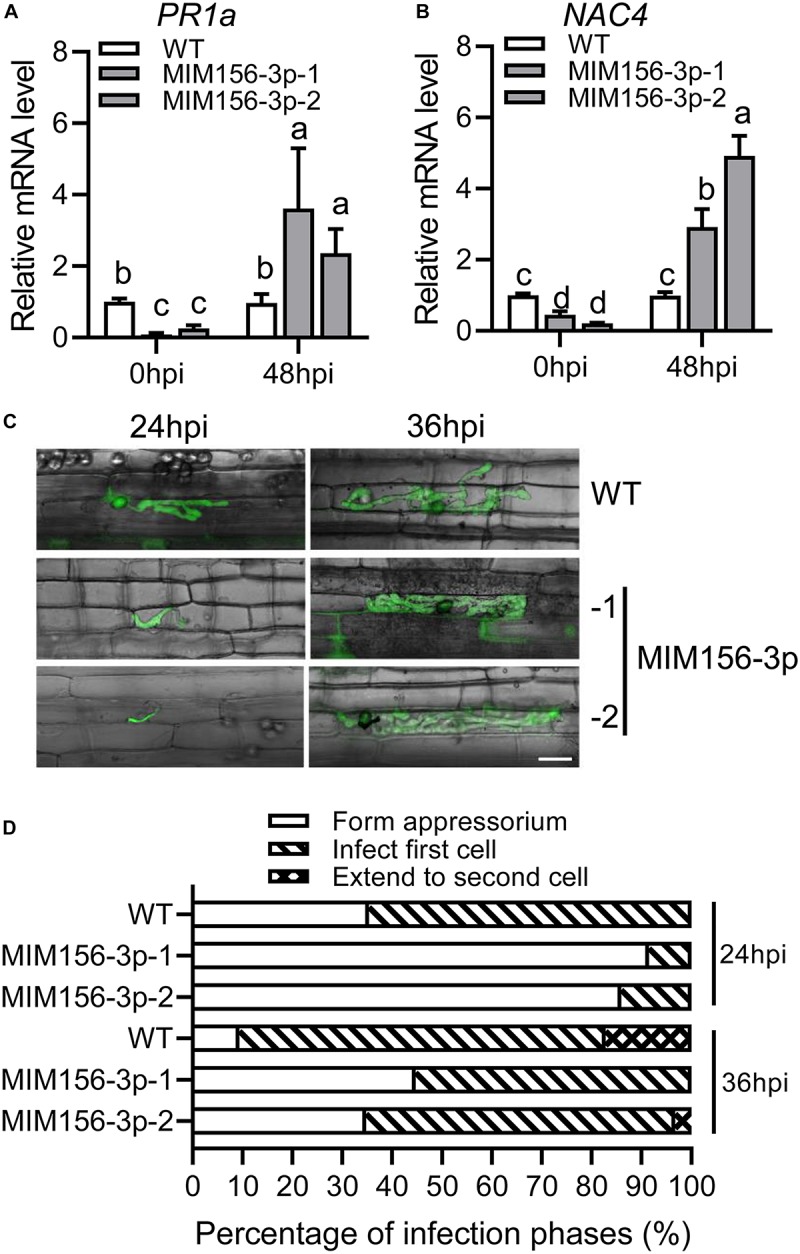
Expression of a target mimic of miR156fhl-3p improves the induction of defense-related genes and delays the *M. oryzae* infection process. **(A,B)** Induction of defense-related genes *PR1a* **(A)** and *NAC4* **(B)** upon *M. oryzae* infection in WT and MIM156-3p lines. Error bars indicate SD (*n* = 3). Different letters above the bars indicate significant differences (*P* < 0.05), as determined by one-way ANOVA analysis followed by *post hoc* Tukey HSD analysis. **(C)** Representative LSCM images show the development of *M. oryzae* strain GZ8 at 24 and 36 hpi in sheath cells of WT and MIM156-3p lines. Scale bars, 20 μm. **(D)** Statistical analysis of *M. oryzae* growth in **(C)**. More than 200 conidia in each line were analyzed. These experiments were repeated two times with similar results.

To understand why blocking miR156fhl-3p leads to compromised susceptibility to blast disease, we examined the expression of the two defense-related response marker genes, *PR1a* and *NAC4*, upon *M. oryzae* strain GZ8 infection. The expression of both genes was induced to significantly higher levels by GZ8 in MIM156-3p than that in control at 48 hpi (Figures 3A,B). Then, we compared the infection process in leaf sheath of WT control and MIM156-3p plants with a LSCM after the inoculation of the GFP-tagged strain GZ8. The growth of GZ8 was delayed in MIM156-3p at 24 and 36 hpi in comparison with that in control (Figure 3C). Quantified infection phase assay showed that more than 60% of spores formed the invasive hyphae in local cells at the infection site in WT, whereas only 10% spores formed invasive hyphae in the local cells in MIM156-3p (Figure 3D). At 36 hpi, more than 70% of hyphae infected into the first cell, and 17% of hyphae extend to the neighbor cells in control. In contrast, about 45% of spores formed the appressorium, and 55% spores formed invasive hyphae and infected into the local cells in MIM156-3p (Figure 3D). These observations suggested that blocking miR156fhl-3p delayed the infection process of *M. oryzae* and promoted the induction of defense-related genes.

### Expression of a Target Mimic of miR156fhl-3p Does Not Penalize Agronomic Traits

**FIGURE 4:**
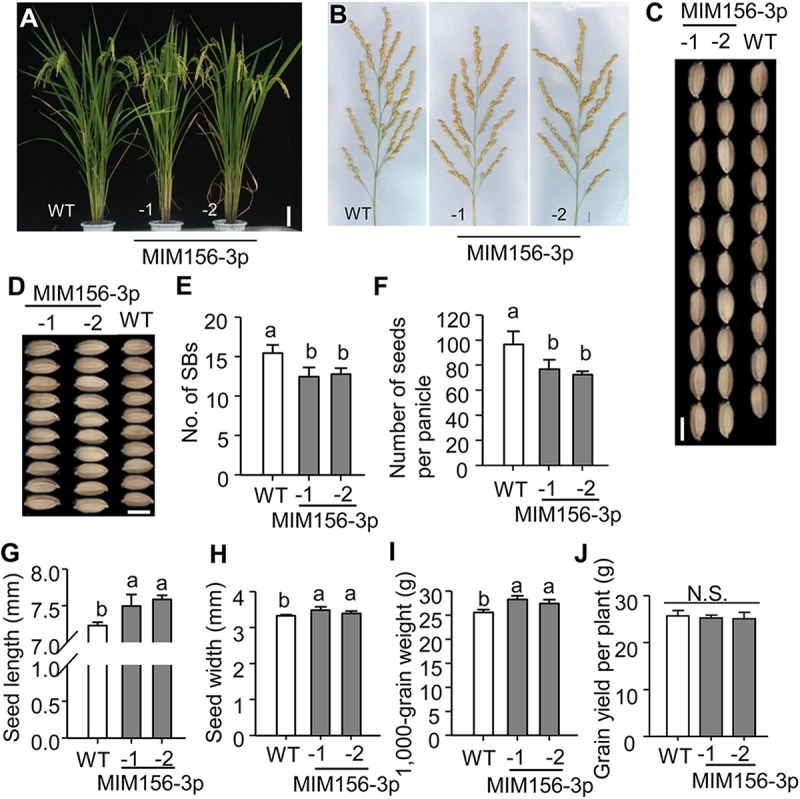
Blocking miR156fhl-3p leads to changed rice agronomic traits without effect on yield. **(A)** Gross morphology of WT and MIM156-3p lines. Size bar, 10 cm. **(B)** Panicle morphology of WT and MIM156 lines. Size bar, 1 cm. **(C,D)** Grain length and width of WT and MIM156-3p. Size bar, 5 mm. **(E–J)** Statistical analysis of the number of secondary rachis branches per panicle (No. of SBs) **(E)**, the number of seeds per panicle **(F)**, seed length **(G)**, seed width **(H)**, 1,000-grain weight **(I)**, and grain yield per plant **(J)** of WT and MIM156-3p lines. Error bars indicate standard deviation (*n* = 5). Different letters above the bars indicate significant differences (*P* < 0.05) determined by one-way ANOVA analysis followed by *post hoc* Tukey HSD analysis.

Activation of blast resistance often penalizes growth and agronomic traits that lead to yield formation (Heil and Baldwin, 2002; Walters and Boyle, 2005; Shimono et al., 2007; Walters and Heil, 2007). To detect whether blocking miR156fhl-3p via target mimicry affected rice growth, we assayed the agronomic traits of MIM156-3p plants. Intriguingly, MIM156-3p showed similar gross plant morphology as WT (Figures 4A,B). Statistical analysis indicated that several agronomic traits were slightly but not significantly decreased in MIM156-3p in comparison with those in WT (Supplementary Figure S3), including plant height, number of tillers per plant, panicle length, and number of primary rachis branches per panicle (No. of PBs). The number of secondary rachis branches per panicle (No. of SBs) was significantly decreased in MIM156-3p plants (Figure 4E), leading to a reduced number of grains per panicle (Figure 4F). In contrast, the seed length (Figures 4C,G) and width (Figures 4D,H) in MIM156-3p were increased, resulting in an increased 1,000-grain weight (Figure 4I). As a result, the grain yield per plant was unchanged in MIM156-3p (Figure 4J) in comparison with that in WT. Altogether, our results indicated that blocking miR156fhl-3p could improve rice blast disease resistance without penalty of yield.

### Expression of a Target Mimic of miR156fhl-3p Suppresses miR156-5p Accumulation and Increases *SPL14* Expression

**FIGURE 5:**
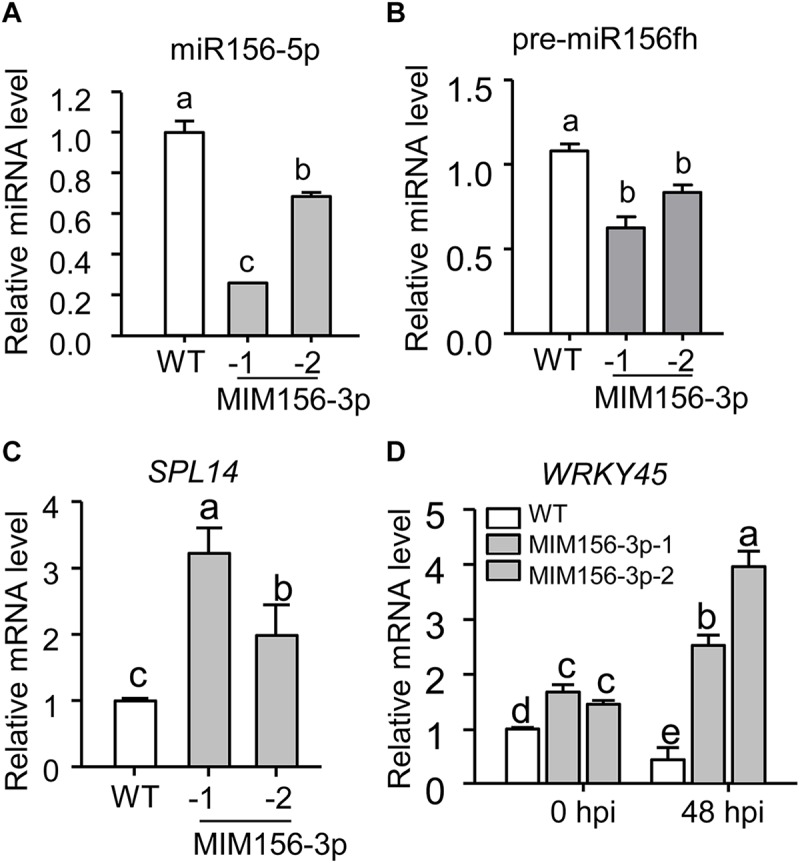
Blocking miR156fhl-3p leads to a decreased abundance of miR156-5p but increased expression of *SPL14* and *WRKY45*. **(A,B)** Relative amount of mature miR156-5p **(A)** and pre-miR156fh **(B)** in WT and MIM156-3p transgenic lines. **(C)** Relative mRNA amounts of *SPL14* in WT and MIM156-3p. **(D)** Relative mRNA amounts of *WRKY45* with or without *M. oryzae* infection in WT and MIM156-3p at indicated time points. In **(A–D)**, error bars indicate SD (*n* = 3). Different letters above the bars indicate significant differences (*P* < 0.05), as determined by one-way ANOVA analysis followed by *post hoc* Tukey HSD analysis. These experiments were repeated two times with similar results.

To uncover how miR156fhl-3p regulated rice resistance against *M. oryzae*, we first predicted potential target genes of miR156fhl-3p by using psRNATarget^2^, an online tool for prediction of miRNA targets. We chose two best-matched candidate genes, namely *LOC_Os02g17280* and *LOC_Os07g37580*, for examination (Supplementary Figure S4A). However, we failed to detect changes in their expressions in both MIM156-3p lines (Supplementary Figures S4B,C), indicating that the two candidates were possibly not regulated by miR156fhl-3p at mRNA level. We then tested whether the expression of MIM156-3p affected the accumulation of miR156-5p. Surprisingly, the abundance of mature miR156-5p was significantly decreased in MIM156-3p in comparison with that in WT (Figure 5A). Consistently, the abundance of pre-miR156fh was also significantly decreased in MIM156-3p in comparison with that in WT (Figure 5B). These data indicate that the blocking of miR156fhl-3p by MIM156fhl-3p could suppress the accumulation of miR156-5p. Previously, miR156-5p was reported to regulate the expression of *SPL14* (Jiao et al., 2010). The SPL14 protein is phosphorylated during *M. oryzae* infection, and the phosphorylated SPL14 binds to the promoter of *WRKY45* to promote its expression. In turn, *WRKY45* positively regulates rice immunity against *M. oryzae* (Shimono et al., 2007; Wang et al., 2018). We speculated that the expression of *SPL14* and *WRKY45* should be altered in MIM156-3p lines. As expected and reversely to the decreased accumulation of miR156, the mRNA amounts of *SPL14* and *WRKY45* were significantly higher in MIM156-3p than in control, and the expression of *WRKY45* was further significantly increased compared to that in control at 48 hpi of *M. oryzae* (Figures 5C,D). These results implied that the mRNA abundance of *SPL14* could be regulated by miR156fhl-3p via interference with miR156-5p, thus boosting the expression of *WRKY45* to enhance rice blast disease resistance.

**FIGURE 6:**
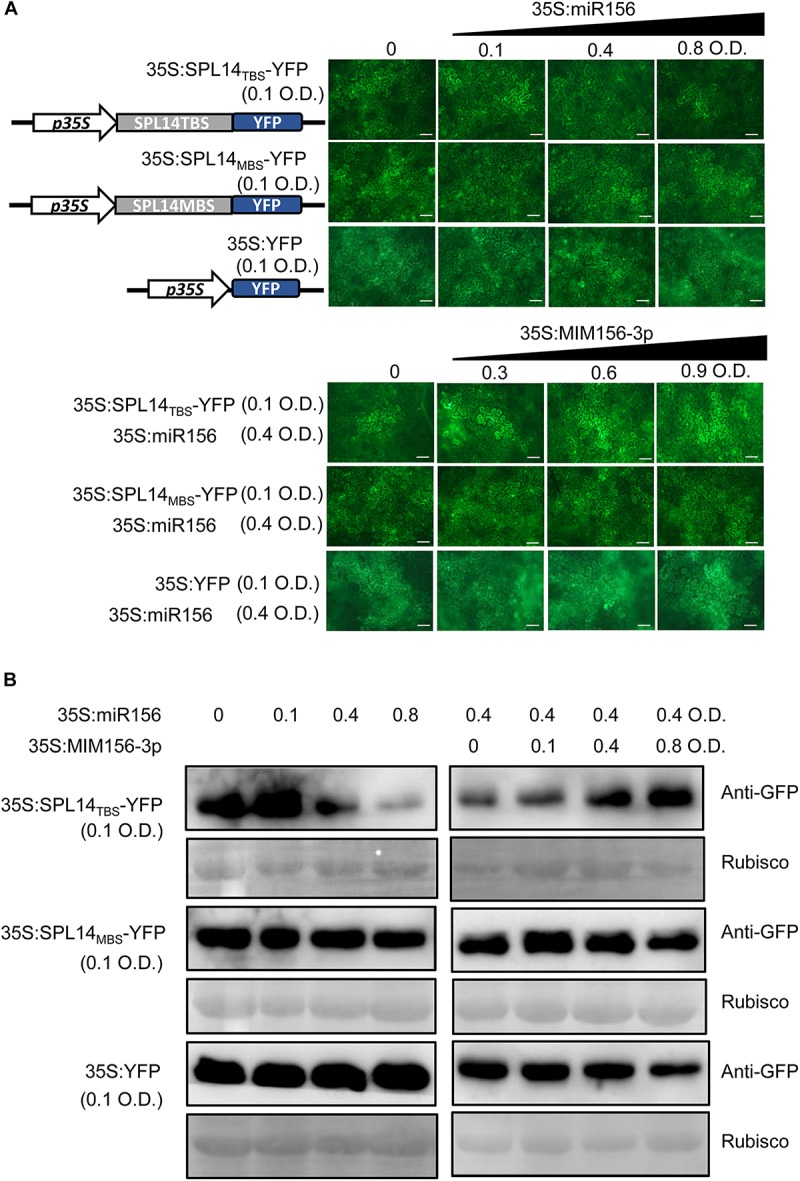
Blocking miR156fhl-3p releases *SPL14* from suppression by miR156-5p. **(A)** Confocal images show the protein levels of SPL14_TBS_-YFP, SPL14_MBS_-YFP, and YFP co-expressed with miR156 or with miR156 and MIM156-3p. The indicated SPL14_TBS_-YFP, SPL14_MBS_-YFP, and YFP reporter constructs were transiently expressed alone or co-expressed with miR156 and MIM156-3p in *N. benthamiana* leaves by Agrobacterium-mediated transformation at the indicated optical density (O.D.) concentration. Size bars, 50 μm. **(B)** Western blotting analysis shows the protein abundance of SPL14_TBS_-YFP, SPL14_MBS_-YFP, and YFP. Protein extracts from infiltrated leaves were collected for Western blot analysis using anti-GFP sera and anti-Rubisco sera, respectively. These experiments were repeated two times with similar results.

To confirm the interference of miR156fhl-3p with miR156-5p, we established a YFP-based reporter system. In the system, we made a construct expressing a YFP reporter that was fused with the miR156 binding site of *SPL14* (SPL14_TBS_) at the N-terminus (SPL14_TBS_-YFP) and a construct expressing a mutant target site of *SPL14* (SPL14_MBS_-YFP). SPL14_TBS_-YFP was separately expressed or co-expressed with miR156 and MIM156-3p in *N. benthamiana* leaves. Then the YFP reporter was examined by YFP intensity and Western blotting. The YFP intensity and protein accumulation were highly accumulated when SPL14_TBS_-YFP was expressed alone. In contrast, the YFP intensity and protein accumulation were significantly decreased when co-expressed with miR156 (Figures 6A,B), indicating suppression of miR156 on SPL14_TBS_-YFP. Intriguingly, the YFP intensity and protein accumulation were recovered when MIM156-3p was added in the co-transient expression (Figures 6A,B). In contrast, the YFP intensity and protein accumulation of SPL14_MBS_-YFP kept unchanged when co-expressed with miR156 or with both miR156 and MIM156-3p (Figures 6A,B). These data imply that MIM156-3p could release the suppression of *SPL14* by miR156 via interference with miR156-5p, leading to activation of *SPL14*.

### miR156 Negatively Regulates Rice Blast Resistance

**FIGURE 7:**
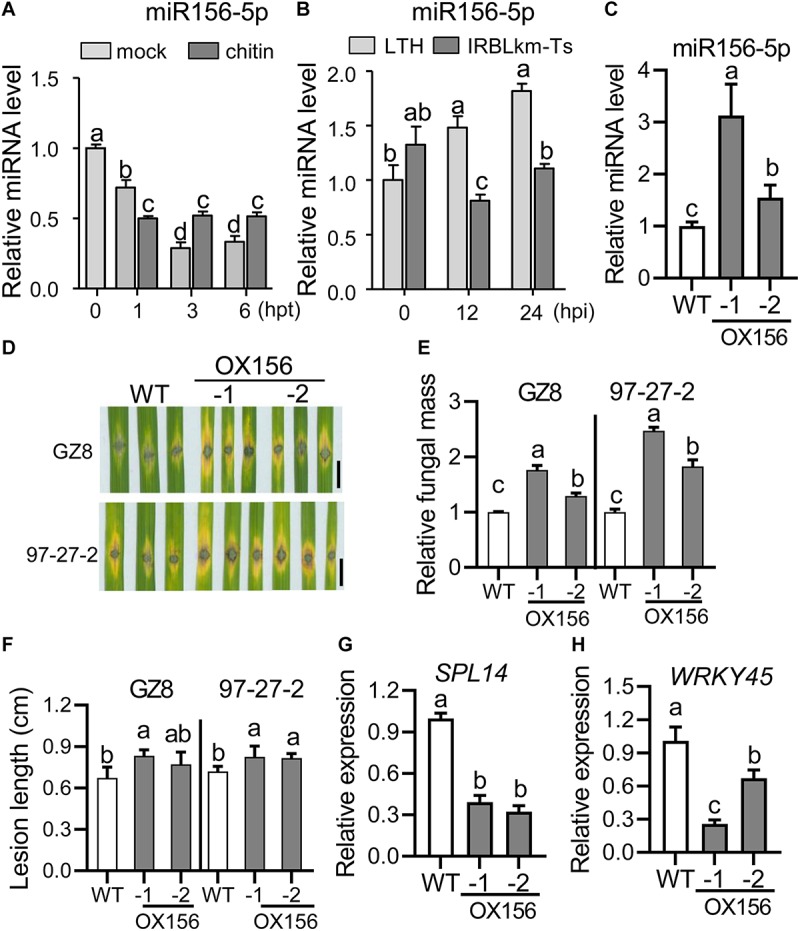
Overexpression of miR156h results in enhanced susceptibility to *M. oryzae.* **(A)** Expression pattern of miR156-5p upon chitin treatment. **(B)** Expression pattern of miR156-5p in LTH and IRBLkm-Ts following *M. oryzae* inoculation. **(C)** Relative mRNA amount of miR156-5p in WT and OX156 lines. RNAs were extracted from leaves of three-leaf-stage plants from the indicated lines. The amounts of mature miR156 were analyzed using Stem-loop RT-qPCR. SnRNA U6 served as the internal reference. **(D)** Disease phenotypes of the indicated lines were observed at 5 dpi of *M. oryzae* strains GZ8 and 97-27-2. Scale bars, 5 mm. **(E)** Relative fungal mass represented by measuring *MoPot2* gene against *OsUbiquitin* DNA level. **(F)** Average lesion length. **(G,H)** RT-qPCR analysis of relative mRNA amounts of *SPL14* **(F)** and *WRKY45* **(G)** in WT and OX156 lines. For **(A**–**C**,**E**–**H)**, error bars indicate standard deviation (*n* = 3). Different letters above the bars indicate significant differences (*P* < 0.05) determined by one-way ANOVA analysis followed by *post hoc* Tukey HSD analysis.

As MIM156-3p plants exhibited enhanced blast resistance via suppressing the accumulation of miR156-5p, we speculated that miR156-5p acts in blast resistance and should be responsive to *M. oryzae*. To confirm this hypothesis, we first examined the expression pattern of miR156-5p following chitin treatment in LTH and following *M. oryzae* invasion in LTH and IRBLkm-Ts. We compared the mock and chitin treatment in inducing miR156 expression. The abundance of miR156-5p was increased at 3 and 6 hpt in comparison with mock treatment (Figure 7A), indicating miR156-5p is responsive to chitin. Moreover, miR156-5p was significantly increased in LTH upon *M. oryzae* infection, whereas it was decreased at 12 and 24 hpi in IRBLkm-Ts (Figure 7B), implying a negative role in pathogen-induced immunity. Then, we constructed transgenic lines overexpressing miR156h (OX156), which formed the same mature sequence as miR156abcdefgij (miR156-5p) (Supplementary Figure S1). OX156 plants showed remarkably decreased plant height (Supplementary Figure S6A) and other agronomic traits (Supplementary Figures S6B–J), as previously reported (Wang L. et al., 2015). We chose two transgenic lines, OX156-1 and OX156-2, for further study (Figure 7C and Supplementary Figure S6A). Both transgenic lines showed enhanced susceptibility to *M. oryzae* strain GZ8 and 97-27-2, with more significant disease lesions (Figures 7D,F) and fungal mass (Figure 7E) in comparison with control via punch-inoculation. The disease lesions in OX156-1 were even bigger than that in OX156-2 plants, which was consistent with the more accumulation of miR156-5p in OX156-1 than that in OX156-2 (Figures 7C–F). Furthermore, we found that the mRNA amounts of *SPL14* and *WRKY45* were decreased in OX156 lines (Figures 7G,H), indicating that miR156-5p could facilitate the growth of *M. oryzae* in a dosage-dependent manner via suppressing the expression of *SPL14*, which in turn down-regulates *WRKY45* expression.

## Discussion

Previously, miRNA-3p was marked as miRNA^∗^ that was considered to be a side-product subjected to decay (Jones-Rhoades et al., 2006; Krol et al., 2010). Later on, miRNA-3p was found to target genes different from those of miRNA-5p and thus performed different fine-tuning functions (Navarro et al., 2006; Zhang et al., 2011). Here, we found that expressing a target mimic of miR156fhl-3p affects the abundance of miR156-5p, which in turn regulates a target gene of miR156-5p to fine-tune the tradeoffs between blast disease resistance and yield traits.

Two techniques provide efficient tools to block the activity of a mature miRNA. One is target mimicry technology that is established based on the modulation of *IPS1* on the miR399 activity in *Arabidopsis*. The other is called short tandem target mimicry (STTM), which exploits two target mimics of a miRNA to increase the efficacy of a target mimic in blocking the function of a miRNA (Yan et al., 2012; Zhang et al., 2017). In our previous study, we found that the abundance of miR156fhl-3p was lower in a resistance accession than in a susceptibility accession; still, its abundance was much more than those of miR156b-3p, miR156cg-3p, and miR156j-3p (Supplementary Figure S2; Li et al., 2014). Here, we exploited target mimicry to explore the function of miR156fhl-3p. Transgenic MIM156-3p plants showed compromised susceptibility to *M. oryzae* with less fungal mass and shorter lesion length than control (Figures 2B–D). Consistently, defense-related genes, such as *PR1a* and *NAC4*, were significantly up-regulated higher in MIM156-3p than in control (Figures 3A,B), and the fungal invasion process was obviously delayed in MIM156-3p plants (Figures 3C,D). Moreover, transgenic MIM156-3p plants also produced a grain yield comparable to that of control, which was built on the orchestration of the number of panicles, panicle size, and grain weight (Figure 4). Therefore, these data indicate that expressing a target mimic of miR156fhl-3p improves blast disease resistance without the yield penalty.

The expression of either a target mimic or an STTM of a miRNA can lead to a decrease in the abundance of the miRNA via an unknown mechanism (Zhang et al., 2017). Here, we found that the abundance of miR156fhl-3p was significantly down-regulated in MIM156-3p lines (Figure 2A). Surprisingly, the abundance of the mature miR156-5p was also significantly down-regulated (Figure 5A), which represents the transcripts from *Osa-miR156a*, *Osa-miR156b*, *Osa-miR156c*, *Osa-miR156d, Osa-miR156e*, *Osa-miR156f*, *Osa-miR156g*, *Osa-miR156h*, *Osa-miR156i*, and *Osa-miR156j* (Supplementary Figure S1). It is well-known that a *MIR* gene is transcribed into a pri-miRNA that is processed into the precursor of a mature miRNA, namely a pre-miRNA (Rogers and Chen, 2013). A pre-miRNA folds into a hairpin that is cleaved by a DCL protein and exported to the cytoplasm, where the DCL processes it into a miRNA-5p/miRNA-3p duplex (Liu et al., 2005). One or both of the strands of the miRNA-5p/miRNA-3p duplex are incorporated into AGO proteins to mediate the silencing of target genes via sequence complementarity (Zhu et al., 2011; Kobayashi and Tomari, 2016). We also found the down-regulation of pre-miR156fh in MIM156-3p lines (Figure 5B), indicating that the target mimic of miR156fhl-3p may interfere with the expression of miR156 family genes at either the transcription or the procession stage. Previously, the expression of *SPL14* in a mutant, i.e., *ipa1* (*Ideal Plant Architecture1*) that carries a mutation at the miR156 target site, was not influenced by the infection of *M. oryzae*; instead, the phosphorylation of IPA1 was changed during *M. oryzae* infection, leading to improvement of both blast disease resistance and yield (Wang et al., 2018). We confirmed such interference of a -3p with -5p using an SPL14_TBS_-YFP-based reporter system, through which we confirmed that the overexpression of a target mimic of miR156fhl-3p interfered with miR156-5p miRNA level, which did release the suppression of miR156-5p on the SPL14_TBS_-YFP accumulation (Figure 6). Consistently, the expression of both *SPL14* and its downstream gene *WRKY45* was increased in MIM156-3p (Figures 5C,D). However, we currently could not tell whether the inference of a -3p with a -5p occurs at the transcription or the post-transcription step. Alternatively, the phenotypes observed in MIM156-3p could be due to altered expression of the target genes of miR156fhl-3p. We searched for the target genes of miR156fhl-3p through website prediction. Among these candidate genes, we selected two genes for expression analysis (Supplementary Figure S4A) and found that their expressions were not significantly altered in MIM156-3p (Supplementary Figure S4B). It is unclear whether the target genes of miR156fhl-3p are involved in regulating rice resistance and agronomic traits. Therefore, future work should focus on searching miR156fhl-3p target genes and exploit Northern blotting to examine the abundance of each pri-miR156 and pre-miR156 to figure out which step is impacted by the target mimic of miR156fhl-3p. Also, future research should focus on addressing whether such interference of a -3p with a -5p is specific to *MIR156* genes or a common phenomenon among different miRNAs.

*MIR156* family genes were classified into two groups based on their functions in regulating rice agronomic straits. Group I includes miR156d, miR156e, miR156f, miR156g, miR156h, miR156i, and miR156j, acting in controlling grain size (Miao et al., 2019); group II includes miR156a, miR156b, miR156c, miR156l, and miR156k, contributing to shoot architecture (Supplementary Figure S5; Miao et al., 2019). Intriguingly, miR156a, miR156b, miR156c, miR156d, miR156e, miR156f, miR156g, miR156h, miR156i, and miR156j are transcribed into the same -5p mature sequences (Supplementary Figure S1). miR156fh is in group I, and miR156l is in group II (Supplementary Figure S5). Transgenic OX156 lines overexpressing miR156h showed dwarfish phenotypes that were similar to previous reports (Supplementary Figure S6; Xie et al., 2006; Dai et al., 2018; Liu et al., 2019). In contrast to MIM156-3p, which exhibited enhanced blast resistance, OX156 showed enhanced susceptibility, which may be due to the down-regulation of *SPL14* and *WRKY45* (Figure 7). Because miR156h represents the major and the most abundant isoform of miR156, these data indicate that miR156 negatively regulates blast disease resistance via the *SPL14-WRKY45* transcription factor module.

**FIGURE 8:**
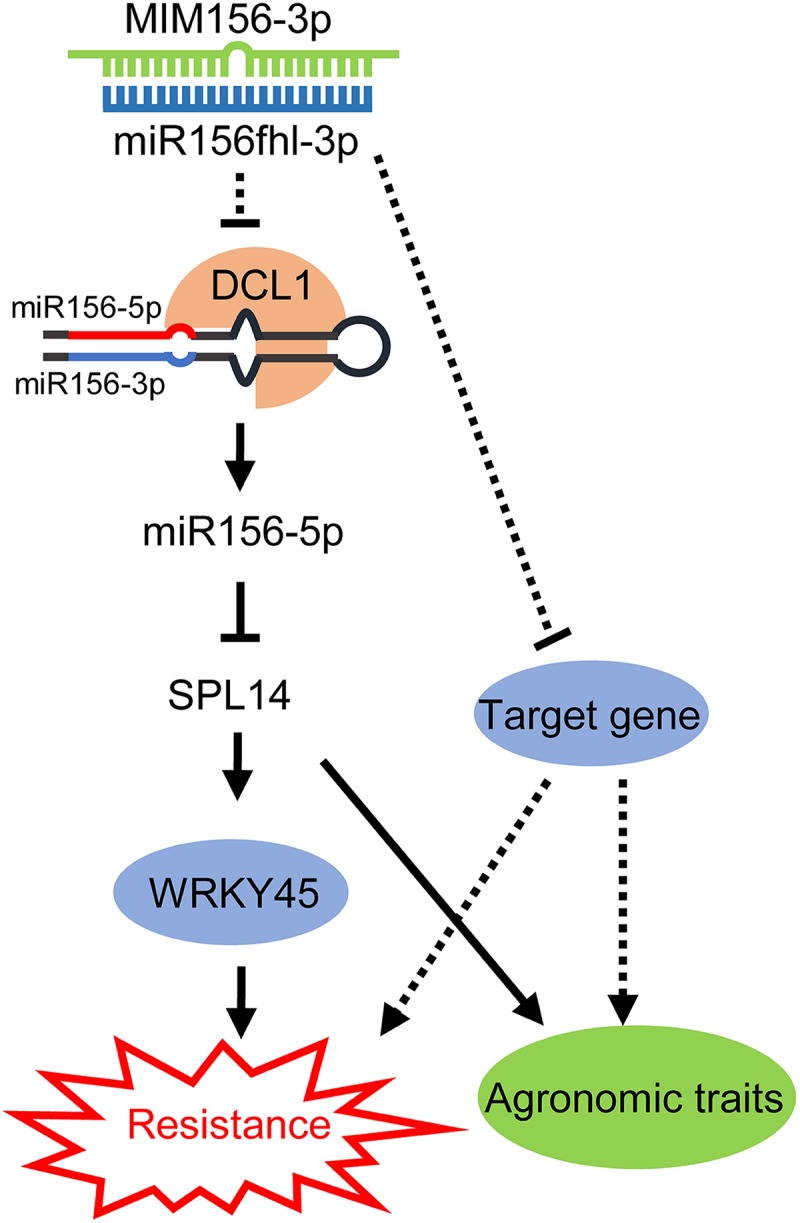
A working model for miR156-3p on regulation of miR156-*SPL14* module in resistance and agronomic traits. Overexpression of a target mimic of miR156fhl-3p interferes with miR156fhl-3p, leading to the down-regulation of miR156-5p via an unknown mechanism, which results in the up-regulation of the miR156-5p target gene *SPL14*. In turn, *SPL14* activates *WRKY45* expression, leading to enhance rice blast disease resistance without fitness cost. Meanwhile, the unknown target genes of miR156fhl-3p might be involved in regulation of blast disease resistance and agronomic traits.

Taken together, we propose a model to summarize our discovery (Figure 8). The expression of a miR156fhl-3p target mimic interferes with the accumulation of both miR156fhl-3p and miR156-5p by an unknown mechanism. Previously, miR156-5p was reported to regulate *SPL14* expression via mRNA cleavage or transcription suppress (Jiao et al., 2010). Overexpression of *SPL14* enhances rice resistance against *M. oryzae* (Wang et al., 2018). Here, miR156-5p was down-regulated in MIM156-3p plants, leading to the up-regulation of *SPL14*, which in turn promotes the expression of WRKY45 to enhance resistance against *M. oryzae*. However, we could not exclude that the target genes of miR156fhl-3p might contribute to rice resistance to *M. oryzae* and be involved in the regulation of agronomic traits from our current data. In conclusion, our findings demonstrate another layer of regulation on *SPL14* expression by miR156 in fine-tuning the tradeoffs between blast disease resistance and yield.

## Data Availability Statement

All datasets generated for this study are included in the article/Supplementary Material.

## Author Contributions

W-MW and YL conceived the study. L-LZ, Y-PZ, HW, S-XZ, L-FW, XY, J-FC, X-PL, and X-CM performed the experiment. J-QZ, MP, and HF carried out the field trial. JF, J-WZ, and Y-YH guided the experimental operation. YL, L-LZ, and W-MW contributed to manuscript writing and editing.

## Conflict of Interest

The authors declare that the research was conducted in the absence of any commercial or financial relationships that could be construed as a potential conflict of interest.

## Footnotes

1 www.mirbase.org

2 http://plantgrn.noble.org/psRNATarget/

## Supplementary Material

FIGURE S1 Schematic diagram of miR156 isoforms and artificial mimicry target of miR156fhl-3p. **(A)** Sequence alignment of the miR156-5p and miR156-3p isoforms. **(B)** Alignment of MIM156h-3p with miR156fhl-3p.

FIGURE S2 RNA-Seq revealed the differential amounts of miR156-3p isoforms following *M. oryzae* inoculation at indicated time points.

FIGURE S3 miR156fhl-3p has no significant effect on several agronomic traits. Statistics data of plant height **(A)**, number of tillers **(B)**, panicle length **(C)** number of primary rachis branches per panicle (No. of PBs) **(D)**, of WT and MIM156-3p lines. Error bars indicate SD (*n* = 5). Different letters above the bars indicate significant differences (*P* < 0.05) determined by one-way ANOVA analysis followed by *post hoc* Tukey HSD analysis.

FIGURE S4 The predicted target genes of miR156fhl-3p are not up-regulated significantly in MIM156. **(A)** Sequence alignments of miR156fhl-3p and predict target sites of indicated genes. **(B,C)** RT-PCR analysis of the predicted target genes expression in WT and MIM156-3p plants. Error bars indicate SD (*n* = 3). Different letters above the bars indicate significant differences (*P* < 0.05) determined by one-way ANOVA analysis followed by *post hoc* Tukey HSD analysis. These experiments were repeated two times with similar results.

FIGURE S5 Phylogenetic tree of rice MIR156 gene based on DNA sequences. 100 bp genomic sequences (20 bp upstream sequences corresponding to mature miR156 and 20 bp downstream sequences) were used to construct the phylogenetic tree.

FIGURE S6 miR156 regulates rice agronomic traits. **(A)** Gross morphology of WT and transgenic plants over-expressing miR156h (OX156). Scale bars, 10 cm. **(B–I)** Statistical analysis of plant height **(B)**, number of tillers **(C)**, panicle length **(D)**, number of primary rachis branches per panicle (No. of PBs) **(E)**, number of second rachis branches per panicle (No. of SBs) **(F)**, number of seeds per panicle **(G)**, seed width **(H)**, seed length **(I)**, 1,000-grain weight **(J)** from the indicated lines. Error bars indicate SD (*n* = 5). Different letters above the bars indicate significant differences (*P* < 0.05) determined by one-way ANOVA analysis followed by *post hoc* Tukey HSD analysis.

TABLE S1 The primers used in this study.
